# Comparative Phosphoproteomics of Classical Bordetellae Elucidates the Potential Role of Serine, Threonine and Tyrosine Phosphorylation in *Bordetella* Biology and Virulence

**DOI:** 10.3389/fcimb.2021.660280

**Published:** 2021-04-13

**Authors:** Laurence Don Wai Luu, Ling Zhong, Sandeep Kaur, Mark J. Raftery, Ruiting Lan

**Affiliations:** ^1^ School of Biotechnology and Biomolecular Sciences, University of New South Wales, Sydney, NSW, Australia; ^2^ Bioanalytical Mass Spectrometry Facility, University of New South Wales, Sydney, NSW, Australia

**Keywords:** *Bordetella pertussis*, *Bordetella*, phosphoproteomics, BvgA, protein phosphorylation, *Bordetella parapertussis*, *Bordetella bronchiseptica*, serine/threonine/tyrosine kinase

## Abstract

The *Bordetella* genus is divided into two groups: classical and non-classical. *Bordetella pertussis*, *Bordetella bronchiseptica* and *Bordetella parapertussis* are known as classical bordetellae, a group of important human pathogens causing whooping cough or whooping cough-like disease and hypothesized to have evolved from environmental non-classical bordetellae. *Bordetella* infections have increased globally driving the need to better understand these pathogens for the development of new treatments and vaccines. One unexplored component in *Bordetella* is the role of serine, threonine and tyrosine phosphorylation. Therefore, this study characterized the phosphoproteome of classical bordetellae and examined its potential role in *Bordetella* biology and virulence. Applying strict identification of localization criteria, this study identified 70 unique phosphorylated proteins in the classical *bordetellae* group with a high degree of conservation. Phosphorylation was a key regulator of *Bordetella* metabolism with proteins involved in gluconeogenesis, TCA cycle, amino acid and nucleotide synthesis significantly enriched. Three key virulence pathways were also phosphorylated including type III secretion system, alcaligin synthesis and the BvgAS master transcriptional regulatory system for virulence genes in *Bordetella*. Seven new phosphosites were identified in BvgA with 6 located in the DNA binding domain. Of the 7, 4 were not present in non-classical bordetellae. This suggests that serine/threonine phosphorylation may play an important role in stabilizing/destabilizing BvgA binding to DNA for fine-tuning of virulence gene expression and that BvgA phosphorylation may be an important factor separating classical from non-classical bordetellae. This study provides the first insight into the phosphoproteome of classical *Bordetella* species and the role that Ser/Thr/Tyr phosphorylation may play in *Bordetella* biology and virulence.

## Introduction

The genus *Bordetella* contains both environmental Gram-negative bacterial species and human pathogens (Gross et al., 2008). *Bordetella pertussis* together with *Bordetella bronchiseptica* and *Bordetella parapertussis*, make up the classical bordetellae group which causes respiratory disease in mammals while non-classical bordetellae species include environmental (*Bordetella petrii*), avian (*Bordetella avium* and *Bordetella hinzii*) and opportunistic human pathogens (*Bordetella holmseii*, *Bordetella trematum* and *Bordetella ansorpii*). The pathogenic classical bordetellae group is hypothesized to have evolved out of free-living environmental non-classical *Bordetella* species (Gross et al., 2008).

Of the three classical species, *B. bronchiseptica* is the ancestral species to *B. pertussis* and *B. parapertussis* (Diavatopoulos et al., 2005). It can survive in the environment and infects a broad range of mammals including dogs, pigs, cats, rabbits and humans (Mattoo and Cherry, 2005). *B. pertussis* and *B. parapertussis* are both host restricted pathogens. *B. pertussis* is responsible for whooping cough (pertussis), a severe, human respiratory disease while *B. parapertussis* contains a human specific lineage which causes pertussis-like disease and an ovine specific lineage which causes pneumonia in sheep (Diavatopoulos et al., 2005).

Genomic studies revealed that *B. pertussis* and *B. parapertussis* both evolved independently from *B. bronchiseptica* and this was associated with large-scale gene-loss (Parkhill et al., 2003). Comparison of the core genome estimates that 51% of the genes are shared by all classical *Bordetella* strains with significant differences found in virulence and surface-protein encoding genes (Park et al., 2012). There are limited proteomic studies that have compared the proteomes of all three classical *Bordetella* species. A recent study by Oviedo et al. (2019) investigated proteomic differences between *B. pertussis* and *B. parapertussis* and identified key expression differences in stress response proteins which may lead to differences in persistence within the host between the two *Bordetella* pathogens.

Despite continued vaccinations with high coverage, whooping cough has re-emerged worldwide in the 21^st^ century with an estimated ~16 million cases every year (World Health Organization, 2010; Xu et al., 2019). This has spurred the need to better understand the biology and virulence behind the causal organism in order to develop more effective vaccines and treatments. One important aspect of pertussis biology is protein phosphorylation, a key post-translational modification which affects a wide range of cellular functions including cell division, metabolism, stress response and virulence (Cummings et al., 2006). Protein phosphorylation in *B. pertussis* has been studied in relation to histidine and aspartate phosphorylation in two-component regulatory systems such as BvgAS (Cummings et al., 2006; Moon et al., 2017), RisAS (Coutte et al., 2016) and the recently described PlrSR (Bone et al., 2017). Two-component systems contain a sensor kinase (e.g. BvgS) and a response regulator (e.g. BvgA). Under specific environmental cues, a histidine residue on the sensor kinase is autophosphorylated and then passed onto an aspartate residue (Asp54) on the response regulator through the histidine-aspartate phosphorylation pathway. This response regulator then binds to DNA and regulates the expression of a number of genes (Boulanger et al., 2013).

In addition to histidine and aspartate phosphorylation in two-component systems, serine, threonine and tyrosine (Ser/Thr/Tyr) phosphorylation, once thought to be unique to eukaryotes, have also been found to occur in other bacterial species (Dworkin, 2015). Bacterial Ser/Thr/Tyr kinases can be divided into two groups: Hanks-type Ser/Thr kinases [or eukaryotic-like serine and threonine kinase (eSTK)] which share a common origin and homology to Ser/Thr kinases found in eukaryotes (Stancik et al., 2018), and bacterial tyrosine kinases (BY-kinases) which share little homology to eukaryotic tyrosine kinases and are typified by Walker’s A and B motifs (Bechet et al., 2009). Tyrosine phosphorylation has been implicated in bacterial virulence with pathogenic bacteria such as *Klebsiella pneumonaie* having a high proportion of tyrosine phosphorylation (25.8%) (Lin M.-H. et al., 2009). Ser/Thr phosphorylation has also been implicated in virulence but are more associated with metabolism and cell division (Pereira et al., 2011). Despite the importance of phosphorylation, the Ser/Thr/Tyr phosphoproteome has not been characterized in *Bordetella* species. Here, we aimed to explore the Ser/Thr/Tyr phosphoproteome of classical *Bordetella* species and examine its role in *Bordetella* biology and virulence.

## Materials and Methods

### Experimental Design and Statistical Rationale

For each *Bordetella* strain used, 3 biological replicates were prepared as described below. For each biological replicate, phosphopeptides were sequentially enriched with TiO_2_ followed by IMAC as described below and one sampling from each fractionation was performed for LC-MS/MS. Statistical differences in the number of phosphorylated serine, threonine and tyrosine residues between the three *Bordetella* strains were determined using Fisher’s exact test in Prism Graphpad (v8.0) with p values < 0.05 assigned as statistically significant. Statistical assessment of essential gene enrichment and enrichment of functional categories between the proteome and phosphoproteome was also determined using Fisher’s exact test and Benjamini-Hochberg adjustment for multiple hypothesis testing correction in Rstudio (v 0.99.903).

### Bacterial Culture and Protein Extraction

*Bordetella* strains (L1423 (*B. pertussis*), RB50 (*B. bronchiseptica*) and 12822 (*B. parapertussis*)) were inoculated into 100 ml Thalen–IJessel (THIJS) media as previously described in Luu et al. (2018b) and incubated for 24 h to reach mid-exponential phase. After 24 h, the cultures were centrifuged, and the resulting cell pellet was washed three times with 25 mM NaHCO_3_. The cell pellet was then resuspended in disruption buffer (50 mM Tris-HCl, 2 mM EDTA and 0.4 mM PMSF) with phosphatase inhibitors (5 mM of each: NaF, β-glycerophosphate, Na Orthovanadate and Na pyrophosphate) and sonicated. The lysate was then centrifuged to remove cellular debris and concentrated using a 3 kDa ultra centrifugal unit (Amicon).

### Protein Digestion and Sequential Enrichment of Phosphopeptides

**Figure 1:**
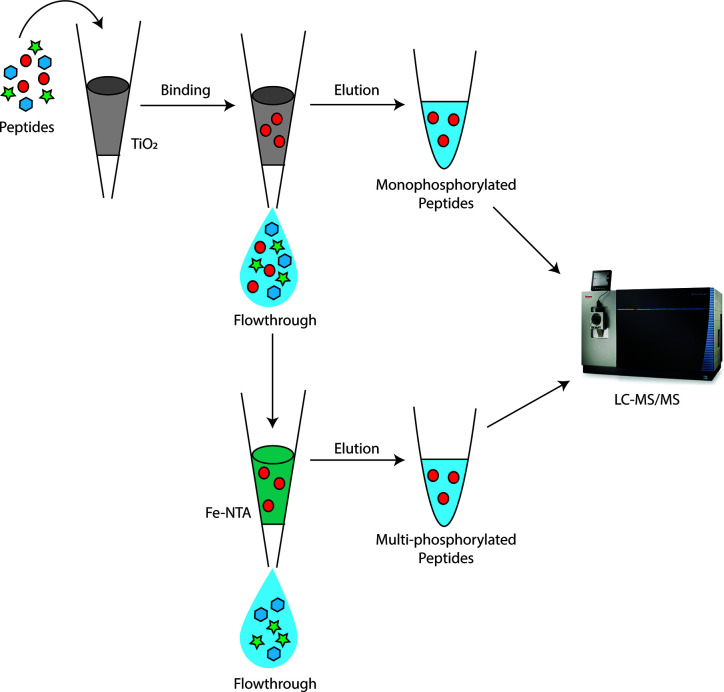
Depiction of workflow for sequential enrichment of *Bordetella* mono and multi-phosphorylated peptides (red circles). Monophosphorylated peptides were first enriched using TiO_2_ beads. The flowthrough from TiO_2_ was then applied to Fe-NTA columns to further separate multi-phosphorylated peptides from non-phosphorylated peptides with IMAC. The elution from TiO2 and IMAC were then analyzed using LC-MS/MS on the Fusion Lumos.

For digestion, 500 µg of proteins were trypsin digested as described in Luu et al. (2017) and cleaned up with an Oasis HLB 1cc vac cartridge (Waters). After cleanup, phosphopeptides were sequentially enriched for monophosphorylated peptides followed by multi-phosphorylated peptides using TiO_2_ and IMAC, respectively (**Figure 1**) (Thingholm et al., 2008). First, TiO_2_ enrichment was performed using Titansphere Phos-TiO Spin Tip kit (3 mg/200 µl) (GL Sciences) according to manufacturer’s instructions. Any uncaptured phosphopeptides in the flow through were cleaned up with an Oasis HLB 1cc vac cartridge and further enriched in a second IMAC fraction using the high-select Fe-NTA Phosphopeptide enrichment kit (ThermoFisher) according to manufacturer’s instructions.

### LC-MS/MS

TiO_2_ and Fe-NTA phosphopeptide enriched samples were each resuspended in 10 µl of 1% formic acid, 0.05% heptafluorobutyric acid and 2% acetonitrile. The samples (2.5 - 5 µl) were then loaded onto the Fusion Lumos (ThermoFisher) connected to an UltiMate nanoRSLC UPLC and autosampler system (Dionex).

The peptides were initially concentrated and desalted with H2O:CH3CN (98:2, 0.2% TFA) at 15 µl/min on a micro C18 precolumn (300 µm x 5 mm, Dionex). After a 4 min wash, the micro C18 precolumn was switched (Valco 10 port UPLC valve, Valco) into line to a fritless nano column (75µ x ~15cm), which contained C18AQ media (1.9µ, 120 Å Dr Maisch). Peptides were then separated on a linear gradient of H2O:CH3CN (98:2, 0.1% formic acid) to H2O:CH3CN (64:36, 0.1% formic acid) at 0.2 µl/min over 30 min. Positive ions were generated with electrospray ionization at 2000V. Data dependent acquisition (DDA) was performed with survey scan from m/z of 350 to 1750, resolution of 120,000 at m/z 200, accumulation target value of 400,000 ions and lockmass enabled (m/z 445.12003). A top-speed approach with a cycle time of 2s was used for data-dependent tandem MS analysis. Ions were fragmented by higher-energy collisional dissociation (HCD) with intensity threshold at 25,000. A mass tolerance of 10 ppm and dynamic exclusion of 20s was set.

### Phosphoproteome Analysis

Raw data files were processed *via* Mascot Daemon (v2.5.1) for peak picking and identification with Oxidation (M), Carbamidomethyl (C), Phospho (ST) and Phospho (Y) set as variable modifications. The remaining search parameters were enzyme: Trypsin, max missed cleavage: 3, mass values: monoisotopic, peptide mass tolerance: 4.0 ppm, MS/MS tolerance 0.4 Da and instrument: ESI-TRAP. For *B. pertussis*, a search database consisting of *B. pertussis* strains: Tohama I (NC_002929.2, 3426 protein sequences), CS (NC_017223.1, 3456 protein sequences), B1917 (CP009751.1, 3458 protein sequences) and B1920 (CP009752.1, 3461 protein sequences) from Genbank were used, while for *B. bronchiseptica* and *B. parapertussis* searches, strains RB50 (NC_002927.3, 4994 protein sequences) and 12822 (NC_002928.3, 4129 protein sequences) were used.

The resulting Mascot files were loaded into Scaffold (v4.11.0) for verification using the ProteinProphet algorithm (Searle, 2010) with protein probability: 99%, peptide probability: 95% and minimum number of peptides: 1. The A-score algorithm (Vincent-Maloney et al., 2011) in Scaffold PTM (v3.3.0) was used to identify and localize phosphosites with the minimum localization confidence set at 95%. All PSM (peptide-spectral matches) for phosphorylation were then manually checked on Scaffold to ensure a prominent b and/or y ion series was present. Each spectrum is available for interpretation using Scaffold-Viewer (see Data availability section). In **Supplementary Tables 1**–**3**, the charge, m/z, MOWSE score for the highest confident PSM is shown. These strict criteria filtered out and eliminated all but the most high quality identification for subsequent analysis.

MPSite (Hasan et al., 2019) and NetPhosBac (v1.0) (Miller et al., 2009) were used to analyze whether serine/threonine phosphosites identified in this study were also bioinformatically predicted to be phosphorylated. Tyrosine prediction was not performed as no bacterial tyrosine phosphorylation prediction tool was available. *Bordetella* phosphosites were also searched against the database of phosphorylation sites in prokaryotes (dbPSP v2.0) (Shi et al., 2020) to identify homologous phosphorylation sites reported in other bacteria.

Protein cellular localization was predicted using PSORTb (v3.0) (Nancy et al., 2010) while functional categories was assigned according to Bart et al. (2010) and Parkhill et al. (2003). Functional enrichment network analysis was performed using STRING (v11.0) (Szklarczyk et al., 2019) for *B. pertussis* and *B. bronchiseptica*. For *B. parapertussis*, functional category enrichment was not performed as *B. parapertussis* was not available in the STRING (v11.0) database.

## Results

### The Phosphoproteome of Classical *Bordetella*

We compared the Ser/Thr/Tyr phosphoproteome of the three classical *Bordetella* species in this study. In *B. pertussis*, the largest number of unique phosphosites was identified with 54 sites (**Supplementary Table 1**). These phosphosites belonged to 53 non-redundant phosphopeptides and 45 proteins (1.3% of total proteins in the genome) (**Table 1**). Of the 54 phosphosites, 72% were located on serine residues (pSer), 17% were on threonine (pThr) and 11% were on tyrosine (pTyr). In *B. parapertussis*, we identified 50 phosphosites from 50 phosphopeptides and 42 proteins (1% of total proteins in the genome) with the distribution being 80% pSer, 12% pThr and 8% pTyr (**Supplementary Table 2**). In *B. bronchiseptica*, 29 phosphosites were identified belonging to 28 phosphopeptides and 23 proteins (0.5% of total proteins in the genome) (**Supplementary Table 3**). The distribution of phosphosites was 69% pSer, 21% pThr and 10% pTyr. The genome size of *B. bronchiseptica* is ~30% and ~17% larger than *B. pertussis* and *B. parapertussis* respectively, however the number of phosphoproteins identified did not reflect this (Parkhill et al., 2003). Additionally, there were no significant differences in the proportion of pSer/pThr/pTyr sites between the three classical bordetellae species.

**Table 1:** Comparison of the phosphoproteome identified in classical *Bordetella* species.

Species	Strain	No. of phosphoproteins	No. of phosphopeptides	No. of phosphosites	pSer (%)	pThr (%)	pTyr (%)
*B. pertussis*	L1423	45	53	54	39 (72%)	9 (17%)	6 (11%)
*B. bronchiseptica*	RB50	23	28	29	20 (69%)	6 (21%)	3 (10%)
*B. parapertussis*	12822	42	50	50	40 (80%)	6 (12%)	3 (8%)

All but one phosphorylated protein (BvgA) identified had 1 or 2 phosphosites. Most peptides were mono-phosphorylated with multi-phosphorylated peptides in classical *Bordetella* uncommon. There were only two phosphopeptides detected that were doubly phosphorylated (AlcA, alcaligin biosynthesis protein and BP3319, choloylglycine hydrolase). The maximum number of phosphorylated sites identified within a single protein in *B. pertussis*, *B. parapertussis* and *B. bronchiseptica* was 5 with BvgA, all of which were located on different mono-phosphorylated peptides.

Of the 120 Ser/Thr phosphosites identified in this study, 67 (56%) were predicted to be phosphorylated by MPSite (Hasan et al., 2019) and/or NetPhosBac (Miller et al., 2009) (**Supplementary Tables 1**–**3**). Of the 133 total phosphosites identified, 31 homologous phosphosites were identified in other bacteria using dbPSP (Shi et al., 2020), including 4 Tyr sites and 2 Ser/Thr sites not predicted by MPSite and NetPhosBac. Therefore, 55% of phosphosites reported in this study were predicted to be phosphorylated or previously identified as phosphosites in other species.

### Common Phosphorylated Proteins in Classical *Bordetella*

**Figure 2:**
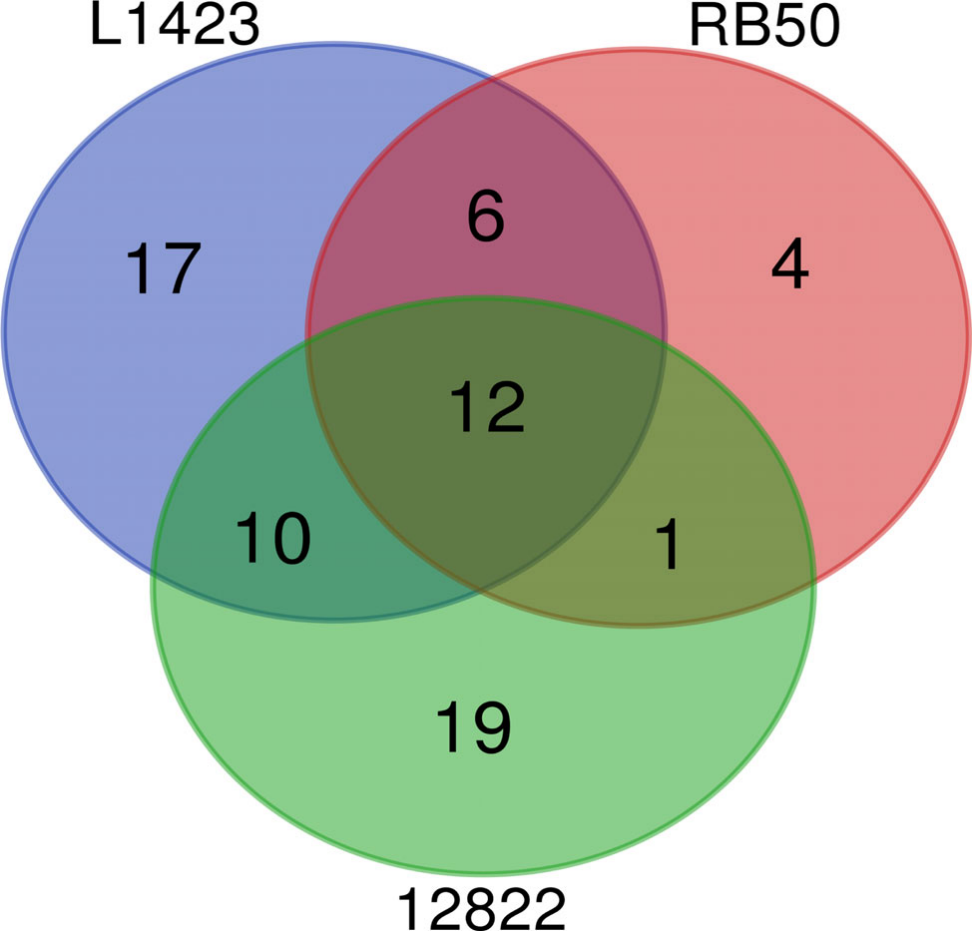
Comparison of the number of phosphorylated proteins identified in *B. pertussis* L1423, *B. bronchiseptica* RB50 and *B. parapertussis* 12822.

In total, 70 phosphorylated proteins were detected across the 3 species. Of the 70 phosphorylated proteins, 29 (41%) were detected to be phosphorylated in two or more classical *Bordetella* species and 12 (17%) were phosphorylated in all three species (**Figure 2**). Of the 12 commonly phosphorylated proteins, 13 phosphopeptides and phosphosites were also shared (**Supplementary Table 4**). Common phosphorylated proteins between the classical *Bordetella* species included 7 metabolism-related proteins: PpsA, SucC, SucD and EftB which are involved in gluconeogenesis/phosphotransferase system for sugar uptake, the TCA cycle and oxidative phosphorylation; AroA, an enzyme in the shikimate pathway for aromatic amino acid biosynthesis; CorC, a magnesium and cobalt transport protein and GlnE which regulates cellular nitrogen levels. The phosphorylation of two proteins involved in stress response (HtpG and Ppk) and one protein in cell wall synthesis (Ddl) were also shared.

**Figure 3:**
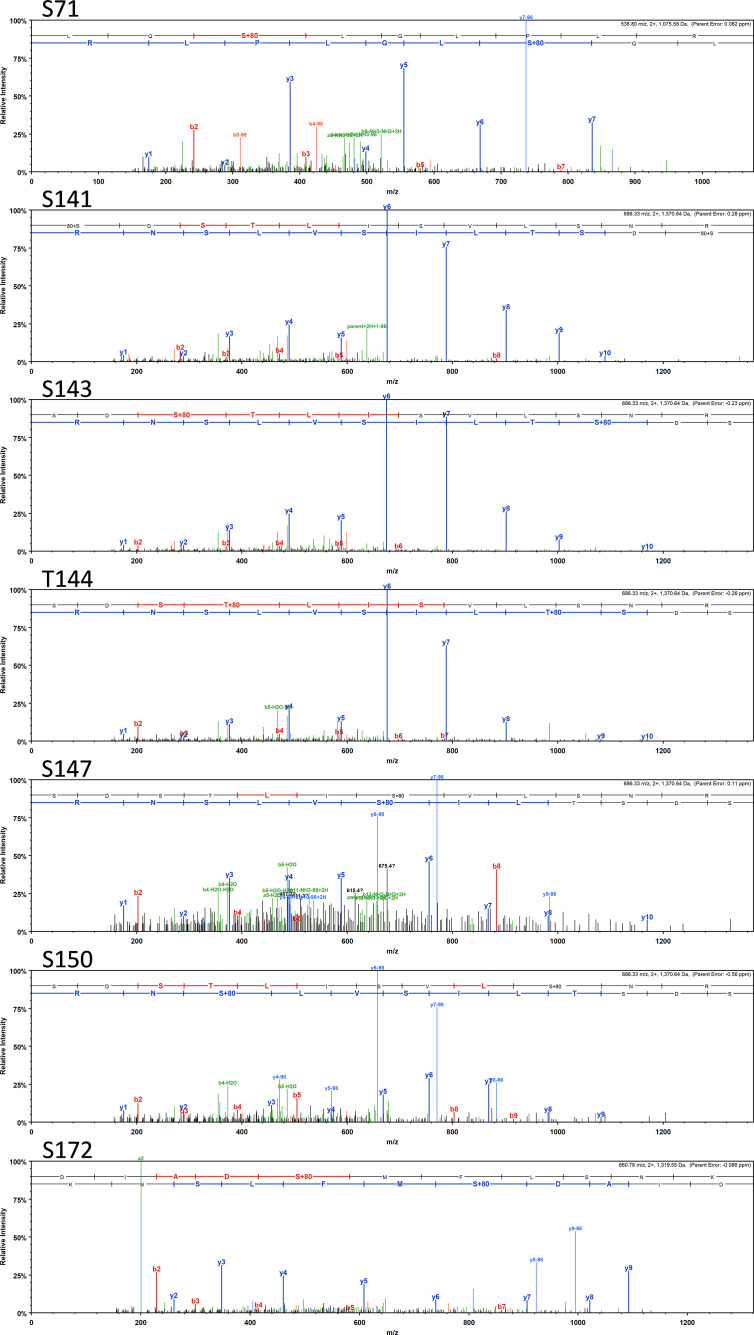
Annotated spectra of the 7 BvgA Ser/Thr sites (Ser71, Ser141, Ser143, Thr144, Ser150 and Ser 172) phosphorylated in classical Bordetellae. Annotated spectra for the highest confident PSM (based on A-score and MOWSE score) is shown.

**Figure 4:**
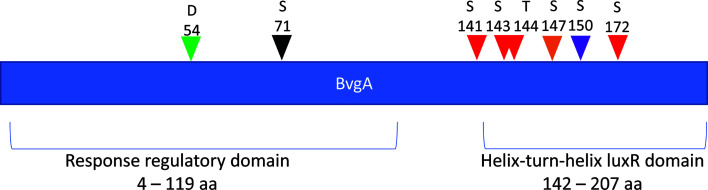
Distribution of phosphosites identified in BvgA. Green arrow denotes the known Asp54 site phosphorylated by BvgS. Red arrows depict Ser/Thr sites phosphorylated in all 3 *Bordetella* species. The black arrow shows a pSer site in *B. pertussis* and *B. bronchiseptica* and the purple arrow illustrates a unique pSer site in *B. parapertussis*. Finally, orange arrows show a unique pSer site in *B. bronchiseptica*.

**Figure 5:**
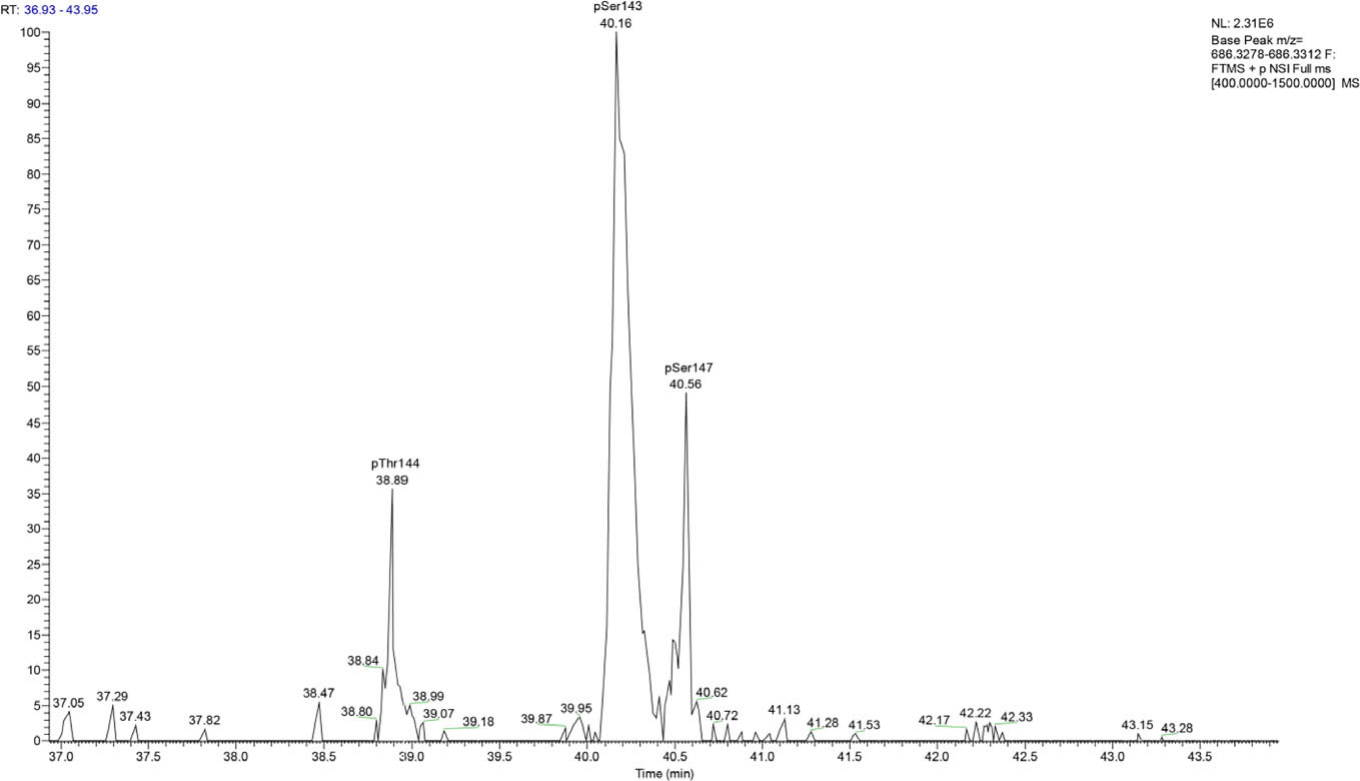
Extracted ion chromatography at m/z 686.3295 ± 2.5 ppm for *B. bronchiseptica* shows three prominent peaks at retention times 38.89, 40.16 and 40.56 which corresponded to BvgA phosphorylation at Thr144, Ser143 and Ser147, respectively.

Most interestingly, BvgA, the BvgAS response regulator and master transcriptional activator for *Bordetella* virulence genes were also commonly phosphorylated in the classical bordetellae group. BvgA was found to be heavily phosphorylated with 7 different Ser/Thr phosphorylation sites identified (**Figures 3** and **4**), 3 of which were identified in all three species (Ser141, Ser143 and Ser 172). A fourth phosphorylated site, Thr144, was identified in *B. pertussis* and *B. parapertussis* while in *B. bronchiseptica*, this phosphorylated site was also identified in the spectra but the A-score localization probability was 93%, slightly lower than the 95% cutoff set. Manual inspection of the spectra helped confirm the phosphorylation of Thr144 in *B. bronchiseptica*. Additionally, different phosphopeptides with the same precursor mass can be readily separated by nanoLC and have different elution times (Santiago et al., 2019). Manual inspection of the extracted ion chromatography at m/z 686.3295 ± 2.5 ppm for *B. bronchiseptica* showed three prominent peaks corresponding to three BvgA peptides phosphorylated at Ser143, Thr144 and Ser147 (**Figure 5**). This provides further evidence that the BvgA peptide (SDSTLISVLSNR) is monophosphorylated at multiple sites. One phosphorylated site (Ser71) was only identified in two species (*B. pertussis* and *B. bronchiseptica*). There was also one unique phosphorylated site in *B bronchiseptica* (Ser147) while another phosphorylated site (Ser150) was unique to *B. parapertussis*. Interestingly, the majority of phosphorylated sites were located at the DNA binding helix-turn-helix luxR domain, toward the C-terminus.

For proteins that were exclusively phosphorylated between two *Bordetella* species (**Supplementary Table 4**), six proteins were commonly phosphorylated in *B. pertussis* and *B. bronchiseptica.* These included a histone protein (BpH1), deoxycytidine triphosphate deaminase (Dcd), two hypothetical proteins (BP2865 and BP1212) and two virulence-related proteins (BfrH and BtrV). BfrH is an iron siderophore receptor while BtrV is an anti-anti sigma factor involved in the regulation of type III secretion system (T3SS) and the only previously characterized serine phosphorylated protein in *Bordetella* (Kozak et al., 2005). Evidence of BtrV phosphorylation at Ser55 was also observed in *B. parapertussis*, however the localization probability A-score was 93%, lower than the 95% cutoff. Based on manual spectra inspection and a previous study, we concluded that BtrV is also phosphorylated in *B. parapertussis*. For *B. pertussis* and *B. parapertussis*, 10 proteins were commonly phosphorylated, many of which are involved in core cellular processes (GpmA, OdhB, PyrH, GyrB, RbfA and BP3130) and responses (GroES, BP0965, BP2757 and BP1012). Similar to BtrV, evidence of GroES phosphorylation at Ser23 was also observed with a localization probability of 88%, marginally lower than the cutoff for confident assignment. Finally, one protein, BP3296 (hydrolase), was commonly phosphorylated *in B. bronchiseptica* and *B. parapertussis*. Besides BtrV and OdhB, all proteins that were exclusively phosphorylated in two *Bordetella* species shared the same phosphopeptide and phosphosites.

### Conservation of Phosphoproteins and Phosphorylation Sites in Classical *Bordetella* Species

Of the 70 phosphorylated proteins in the classical bordetellae group, only 1 gene (BPP4111) was absent in *B. pertussis* while another was a pseudogene (BP0021) and is not expressed. Therefore, there were 68 phosphorylated proteins (97%) that were classified as “core proteins” in classical *Bordetella* with the potential to be phosphorylated.

To determine whether phosphorylation sites identified in one *Bordetella* species were conserved in other classical *Bordetella* species, we mapped the phosphorylation site and 6 amino acids sequences on either side (termed: phosphosite sequence) to the corresponding homologous proteins in the remaining two species. The phosphorylation site and adjacent amino acid sequences were highly conserved with 96-100% of phosphosite sequences showing 100% identity between homologous genes in classical *Bordetella* species.

Finally, we also examined if phosphorylation differences between classical *Bordetella* species may be due to proteome differences. We compared whether the phosphoproteins identified in this study were detected in the proteomes from previous *Bordetella* studies (Kurushima et al., 2012; Luu et al., 2018a; Luu et al., 2018b; Oviedo et al., 2019). Most phosphoproteins (70-75%) identified in one *Bordetella* species have been previously detected in the proteome of other *Bordetella* species (**Supplementary Table 5**).

### Essentiality of Phosphorylated Proteins

In a previous study, 609 genes out of 3624 genes (16.8%) in Tohama I were classified as essential when grown *in vitro* (Gonyar et al., 2019). We found 23 (51.1%) of the 45 phosphorylated proteins in *B. pertussis* fell into the essential genes, suggesting a higher proportion of essential genes that are phosphorylated (Fisher’s exact test, p < 0.0001). This finding highlights the importance of phosphorylation in *B. pertussis*.

### Functional Category Analysis of Phosphorylated Proteins

**Figure 6:**
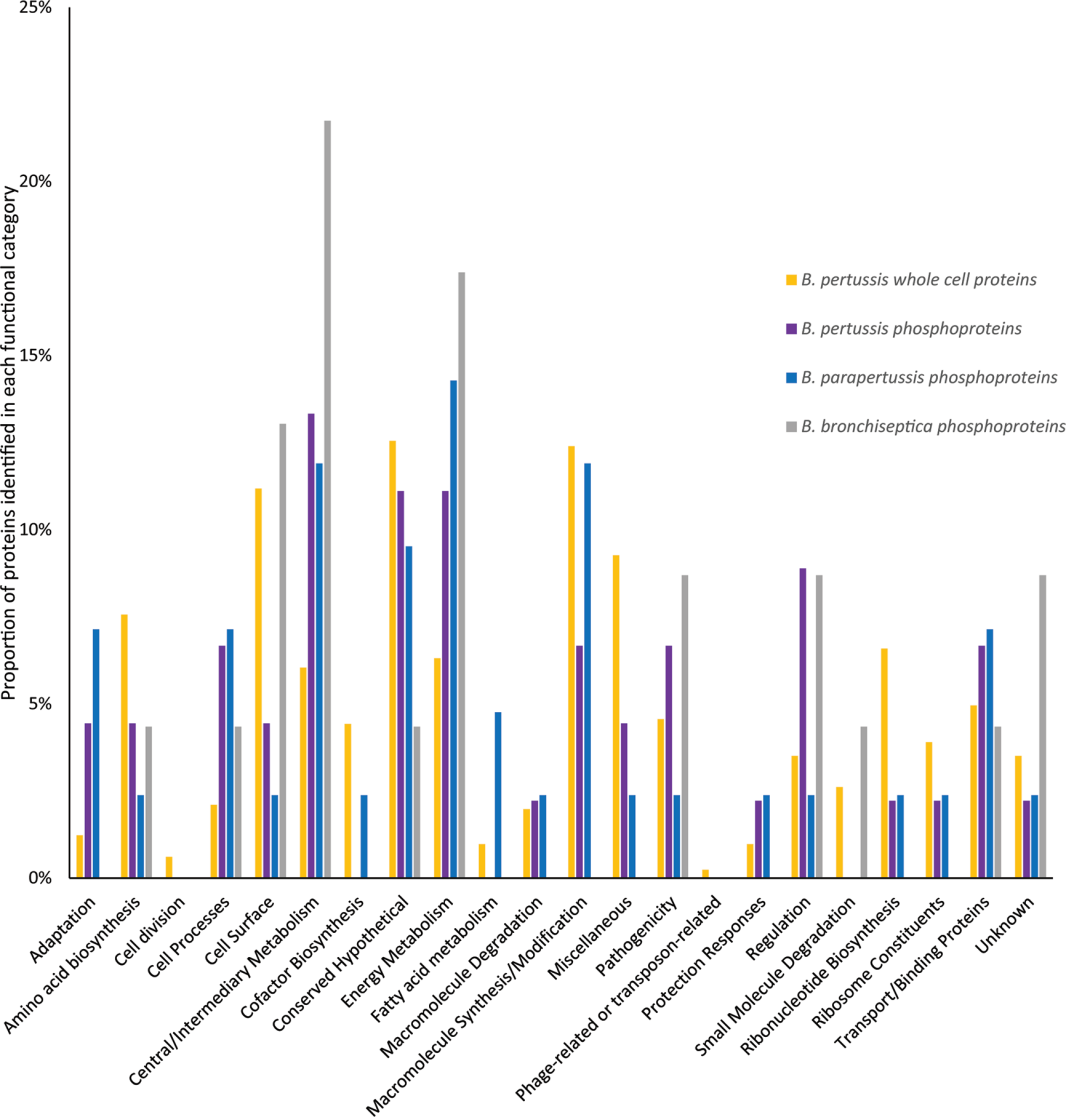
Comparison of the functional categories of phosphorylated proteins identified in *B. pertussis* (purple bars) and whole cell *B. pertussis* proteins identified in Luu et al. (2018a; 2018b) (blue bars).* denotes significant difference between phosphorylated and whole cell proteins.

Functional category distribution and cellular localization of phosphorylated proteins were similar between the 3 species. The majority of phosphorylated proteins were cytoplasmic (52%-74%), followed by cytoplasmic membrane (13%-14%) and outer membrane (0–4%) while the cellular localization for 12-30% of proteins were unknown. The primary functional categories identified for phosphorylated proteins were central/intermediary metabolism (12%-22%), energy metabolism (11%-17%), conserved hypothetical proteins (4%-11%), cell surface (2%-13%) and macromolecule synthesis/modification (0%-12%) (**Figure 6**). This was followed by transport/binding protein (4%-7%), cell process (4%-7%) regulation (2%-9%), and pathogenicity (2%-9%). Compared to whole cell proteins identified in our previous *B. pertussis* proteomic studies (Luu et al., 2018a; Luu et al., 2018b), there was a higher proportion of regulation and central/intermediary metabolism proteins in the *B. pertussis* phosphorylation dataset, although this was not statistically significant (**Figure 6**).

A functional enrichment network analysis of phosphorylated proteins with STRING identified 12 and 11 significantly enriched molecular function GO terms in *B. pertussis* and *B. bronchiseptica* (**Supplementary Table 6**). Enriched GO terms were primarily related to binding including ATP/nucleotide binding, ion binding and protein/unfolded protein binding. Enriched local network clusters and enriched UniProt keywords in STRING also identified an enrichment of clusters/keywords related to carbon and nitrogen metabolism such as the TCA cycle and pentose phosphate pathway (**Supplementary Table 6**).

**Figure 7:**
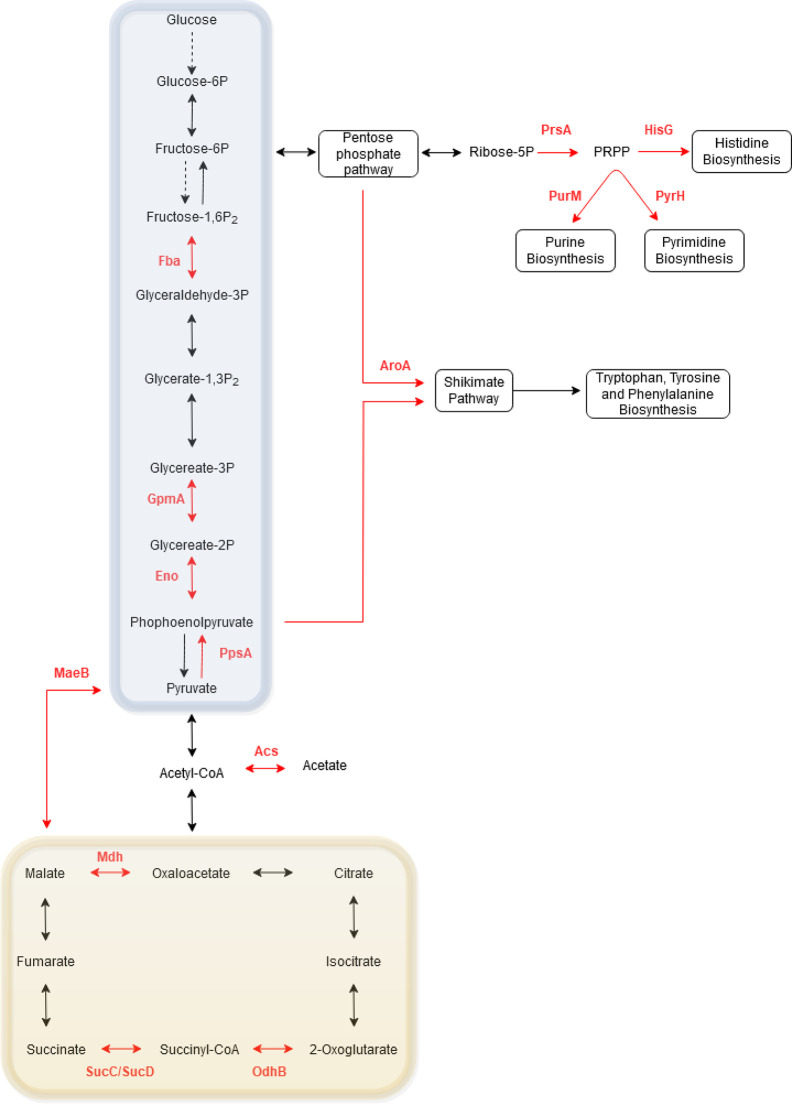
Schematic of the phosphorylated proteins involved in *Bordetella* central carbon and amino acid metabolism. Red arrows and text illustrate phosphorylated proteins and their enzymatic pathway in *Bordetella*. Dashed arrows illustrate enzymes that are absent in *Bordetella*. The glycolysis/gluoconeogenesis pathway is highlighted in the blue box while the TCA cycle is shown in the yellow box.

To further elucidate the role of phosphorylation in metabolism, the phosphoproteins identified in one or more *Bordetella* species were collated and mapped onto KEGG pathways (**Figure 7**). Of the *Bordetella* proteins involved in central carbon metabolism: four enzymes (Fba, GpmA, Eno and PpsA) in the glycolysis/gluconeogenesis pathway and four enzymes in the TCA cycle (OdhB, SucC, SucD and Mdh) were phosphorylated. PrsA, an enzyme that catalyzes synthesis of phosphoribosyl pyrophosphate (PRPP), was also phosphorylated. PRPP is an important intermediary metabolite required for histidine and nucleic acid biosynthesis.

Two enzymes required for the biosynthesis of amino acids (HisG for histidine and AroA for tryptophan, tyrosine and phenylalanine) and two enzymes for nucleotide synthesis (PurM for purines and PyrH for pyrimidines) were also phosphorylated. Finally, one enzyme involved in acetate (Acs) and pyruvate (MaeB) metabolism pathway was phosphorylated.

### Phosphorylated Virulence Factors in *Bordetella*

As stated above, BtrV (the T3SS anti-anti sigma factor) and BvgA (the virulence response regulator) were the only virulence factors commonly phosphorylated across all 3 *Bordetella* species (**Table 2**). In addition, four other virulence factors were also phosphorylated in one or more *Bordetella* species (**Table 2**). Three were involved in iron acquisition including BfrH, a siderophore receptor (Burgos et al., 2010), as well as AlcA and AlcC, both of which are involved in the synthesis of the alcaligin siderophore. BfrH was phosphorylated in both *B. pertussis* and *B. bronchiseptica* while AlcA and AlcC was only phosphorylated in *B. pertussis* and *B. parapertussis* respectively. The final phosphorylated virulence factor was BcrH2, a T3SS chaperonin protein for the pore forming proteins BopB-BopD (Nogawa et al., 2004). BcrH2 was phosphorylated only in *B. pertussis*.

**Table 2:** Phosphorylated virulence factors in classical *Bordetella* species.

Virulence factor	Protein symbol	Virulence pathway	*B. pertussis*	*B. bronchiseptica*	*B. parapertussis*
Virulence factors transcription regulator	BvgA	BvgAS master virulence regulator	S71, S141, S143. T144 & S172	S71, S141, S143, S147, S172 & T144	S141, S143, T144, S150 & S172
Anti-sigma factor antagonist	BtrV	T3SS	S54 & S55	S54	S55
	BcrH2	T3SS	S148	–	–
Ferric siderophore receptor	BfrH	Iron acquisition	T195	T195	–
Alcaligin biosynthesis enzyme	AlcA	Iron acquisition	S158 & Y161	–	–
Alcaligin biosynthesis enzyme	AlcC	Iron acquisition	–	–	T139

**Figure 8:**
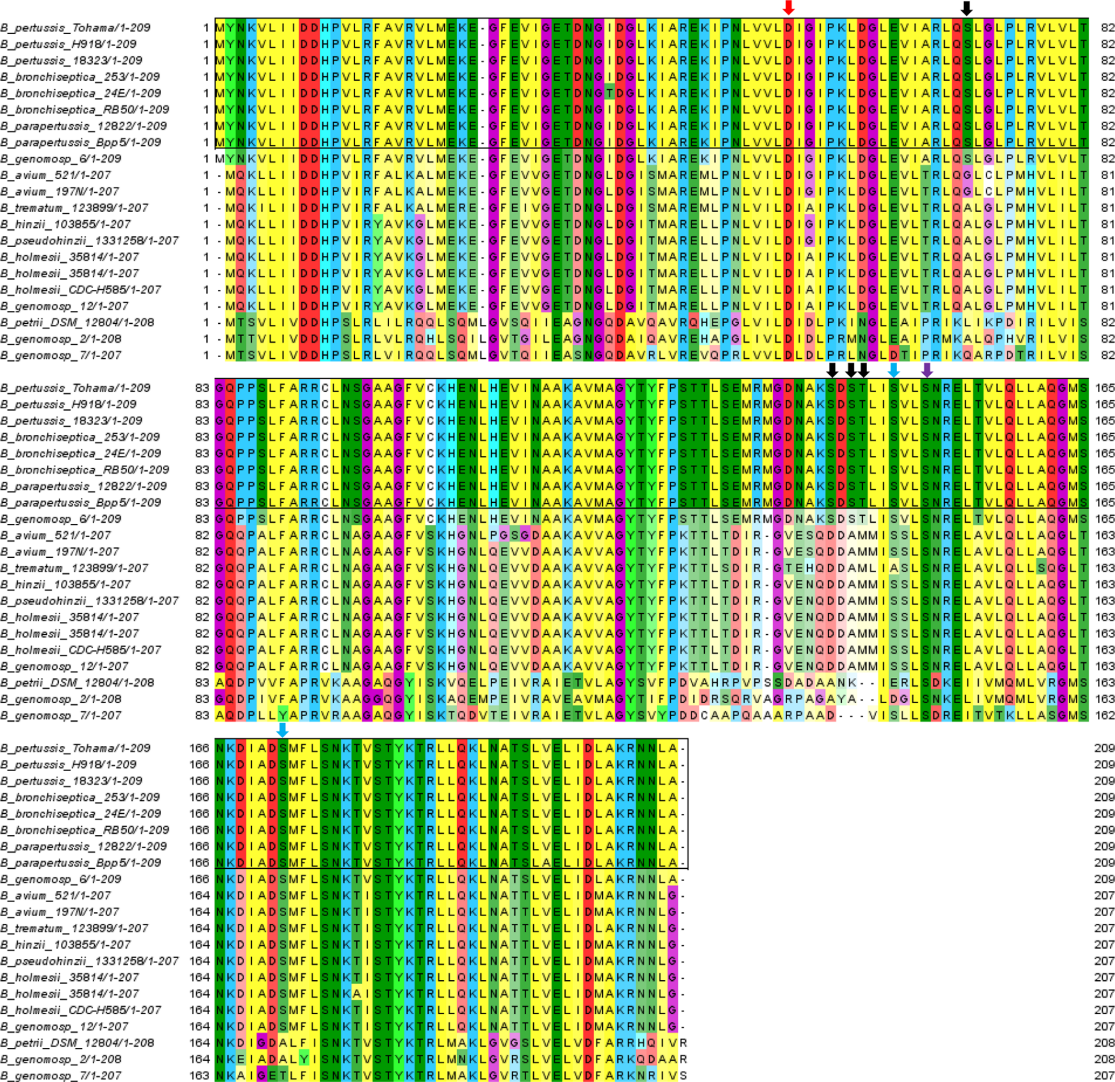
Multiple sequence alignment of BvgA sequences in classical and non-classical *Bordetella* species. The red and purple arrow shows the phosphorylated Asp54 and Ser150 site conserved in all *Bordetella* species, respectively. Black arrows illustrate pSer/pThr sites (Ser71, Ser141, Ser143 and Thr 147) conserved in classical and absent in non-classical *Bordetella*. Blue arrows illustrate pSer sites (Ser147 and Ser172) conserved in most *Bordetella* species. Classical *Bordetella* species are depicted at the top in the black outline while non-classical *Bordetella* species are below.

To further compare the conservation of identified BvgA phosphosites in pathogenic classical and non-classical environmental/opportunistic *Bordetella* species, all available *Bordetella* BvgA amino acid sequences from UniProt (up to 1^st^ of October 2020) were downloaded and aligned using MUSCLE (Edgar, 2004) and MEGA X (Kumar et al., 2018). Sequence alignment showed that the previously characterized aspartate phosphorylation site (Asp54) was conserved in 100% of *Bordetella* species (**Figure 8**). Of the 7 newly identified Ser/Thr sites in this study, only 1 site (Ser150) was also conserved in every *Bordetella* species. Two sites (Ser147 and Ser172) were present in all classical *Bordetella* and most non-classical *Bordetella* species, with Ser172 absent in *B. petrii* and *B.* genomospecies 2 and 7 while Ser147 was also absent in the aforementioned species and *B. trematum*. Interestingly, 4 phosphorylation sites (Ser71, Ser141, Ser143 and Thr144) were conserved and unique to all classical *Bordetella* species and absent in non-classical *Bordetella* (**Figure 8**).

### Potential Ser/Thr/Tyr Kinases in *Bordetella*

To identify potential *Bordetella* Hanks-type Ser/Thr kinases (BstK), we searched the SignalCensus database (Galperin et al., 2010) and NCBI conserved domain database [for the Pkc_like superfamily (cl21453)] (Marchler-Bauer et al., 2017). In the SignalCensus database, four potential Ser/Thr kinases were identified in *B. bronchiseptica* and *B. parapertussis*, and three in *B. pertussis* (**Table 3**). Two of the potential kinases identified in all 3 species were UbiB, a potential protein kinase regulator of UbiI which is involved in ubiquinone biosynthesis, and BP2349, a 3-deoxy-D-manno-octulosonic-acid kinase which may be involved in the LPS core biosynthesis pathway (White et al., 1999; Poon et al., 2000). Interestingly, BP0018 and BB0818 are uncharacterized membrane proteins with a Pkc_like domain, present in Ser/Thr kinases. BP0018 is present in all three species but BB0818|BPP0732 is only present in *B. bronchiseptica* and *B. parapertussis*. Both proteins may be potential BstK. In the NCBI conserved domain database (cl21453), a further three potential kinases were identified in all three species. BP2792 was a hypothetical protein while BP2342 and BP3672 both belonged to the subfamily: PhoP regulatory network protein YrbL which are important regulators of adaptation to low Mg^2+^ environments and virulence (Zwir et al., 2005).

**Table 3:** Potential Ser/Thr/Tyr kinases identified in *Bordetella*.

*B. pertussis*	*B. bronchiseptica*	*B. parapertussis*	Annotation
BP0018	BB0017	BPP0017	Putative membrane protein (PKc_like domain)
BP0164	BB4465	BPP3992	Ubiquinone biosynthesis protein UbiB
BP2349	BB3418	BB3418	3-deoxy-D-manno-octulosonic-acid kinase
	BB0818	BPP0732	Putative membrane protein (PKc_like domain)
BP2792	BB1918	BPP2471	Hypothetical protein
BP2342	BB3411	BPP1697	PhoP regulatory network protein YrbL
BP3672	BB0087	BPP0088	PhoP regulatory network protein YrbL

To identify BY-kinases in *Bordetella*, the BYKdb (Jadeau et al., 2012) and NCBI conserved domain database [BY-kinase domain (cd05387)] (Marchler-Bauer et al., 2017) were searched. No bacterial tyrosine kinases (BY-kinase) were identified in the classical *Bordetella* group in BYKdb and in the NCBI conserved domain database entry for BY-kinase domain (cd05387). One potential BY-kinase (protein accession no: WP_066122743.1) in BYKdb was identified in some non-classical *Bordetella* species (*B. ansorpii*, *B. bronchialis*, *B. flabilis*, *Bordetella* sp. N, H567 and HZ20, *Bordetella* genomosp. 8, 9, 10, 11 and 13).

## Discussion

Protein phosphorylation is an important post translation modification and a rapid signaling mechanism used by cells in response to different environmental changes. Most previous phosphoprotein studies of *Bordetella* species have predominantly focused on the aspartate and histidine phosphorylation of the two-component systems such as BvgAS (Cummings et al., 2006), RisAS (Coutte et al., 2016) and PlrSR (Bone et al., 2017). Although a previous bacterial phosphoproteomic study (Lai et al., 2017) was able to identify several pHis and pAsp sites, we could not confidently verify any spectra which supported pHis and pAsp sites when Phospho (H) and Phospho (D) were included as variable modifications in our Mascot searches. This was presumably because of the instability of these modifications in acidic buffers. Therefore, pHis and pAsp were not analyzed further. This study did identify 45, 42 and 23 Ser/Thr/Tyr phosphorylated proteins in *B. pertussis*, *B. parapertussis* and *B. bronchiseptica*, respectively. Functional categories of phosphorylated proteins were diverse and encompassed key regulatory, housekeeping and metabolic proteins. Most previous bacterial phosphoproteomic studies in non-*Bordetella* species used 1– 100 milligrams of protein and employed laborious SCX fractionation steps (separating up to 15 fractions), which greatly increases costs and run time (**Supplementary Table 7**) (Manteca et al., 2011). Our use of sequential phosphopeptide enrichment (**Figure 1**) coupled to a highly sensitive Fusion Lumos mass spectrometer enabled identification of similar numbers of phosphoproteins using 0.5 milligram of initial protein input. The advantage of requiring less input protein makes bacterial phosphoproteomics more feasible and accessible, especially for slow growing and fastidious bacteria such as *B. pertussis*.

### Extent and Conservation of Ser/Thr/Tyr Phosphorylation in the Classical *Bordetellae*

This study found that Ser/Thr/Tyr phosphorylation was highly conserved across the classical bordetellae group and controlled similar core and essential metabolic and virulence pathways in different species. The evolution of *Bordetella* from environmental species to host adapted pathogen is associated with progressive genome reduction. However, despite significant gene loss in *B. pertussis* and *B. parapertussis*, phosphoproteins were highly conserved with only two phosphorylated proteins not found (absent/pseudogene) in all 3 classical species. The strong conservation despite gene loss suggests a strong selection pressure for phosphorylation and that Ser/Thr/Tyr phosphorylation play an important role in regulating fundamental cellular processes in *Bordetella* including transcription, translation, cell division, stress response, transport and metabolism.

Additionally, there was a high overlap of common phosphorylated proteins (41%) detected between two or more species and a similar proportion of pSer (~70%)/pThr (~20%)/pTyr (10%) sites. Almost all phosphosite sequences identified in one *Bordetella* species were conserved in other *Bordetella* species. Since only one hypothesized Ser/Thr/Tyr kinase was identified to be missing in *B. pertussis* (BB0818|BPP0732) (**Table 3**), this indicates that phosphosites identified in one *Bordetella* species also have the potential to be phosphorylated in other *Bordetella* species.

Remarkably, despite being distantly related, the proportion of phosphorylated pSer/pThr/pTyr sites in *Bordetella* were similar to those observed in other Gram-negative and Gram-positive model organisms such as *E. coli* K-12 and *B. subtilis* (Macek et al., 2007; Macek et al., 2008) (**Supplementary Table 7**). Many of the phosphoproteins identified in this study, especially those related to central carbon and amino acid metabolism (Fba, GlmM, Eno, SucC and AroA etc.) were also phosphorylated in other diverse bacterial species when these pathways were functional (Shi et al., 2020). This provides further evidence of the ubiquitous nature of Ser/Thr/Tyr protein phosphorylation in bacteria and the important role it plays in providing another layer of control to the cell to rapidly regulate metabolic and housekeeping proteins as phosphorylation is the fastest mode of regulation (Yang et al., 2019).

Many phosphoproteins were only detected in a single *Bordetella* species. It is possible that phosphosites identified in one *Bordetella* species may also have been phosphorylated in other *Bordetella* species but were missed due to limitations in instrument sensitivity to detect the phosphosite. It is also possible that the same phosphosite in different species may have different occupancy levels and hence were not detected in other species where the phosphosite occupancy may be lower. Finally, it is possible that the wide variation in phosphoproteins and sites identified in this study may reflect the highly dynamic nature of protein phosphorylation, the presence of other unknown regulators controlling kinases/phosphatases or the different growth stages of the three species.

### Ser/Thr/Tyr Phosphorylation May Regulate Central Carbon and Nitrogen Metabolism

*Bordetella* proteins involved in central carbon and nitrogen metabolism were found to be significantly enriched for phosphorylation including 15 enzymes involved in glycolysis/gluconeogenesis, TCA cycle, amino acid and nucleotide synthesis (**Figure 7**). The classical bordetellae group does not contain a functional glycolysis pathway due to the absence of key genes (glucokinase, phosphofructokinase and fructose-1,6-bisphosphatase) (Parkhill et al., 2003), however it does contain a functional and essential gluconeogenesis pathway (Gonyar et al., 2019). Four enzymes in this pathway were found to be phosphorylated which suggests that the gluconeogenesis pathway in *Bordetella* may be under the control of Ser/Thr/Tyr phosphorylation. In addition to gluconeogenesis, several enzymes in the TCA cycle were also observed to be phosphorylated. *Bordetella* was long thought not to possess a complete functional TCA cycle due to its inability to metabolize acetyl-CoA and oxaloacetate to 2-oxoglutarate (Thalen et al., 1999). However recently, several studies have confirmed that *Bordetella* does contain a complete functional and essential TCA cycle (Izac et al., 2015; Gonyar et al., 2019). Although the *Bordetella* genomes contain all the necessary genes for the TCA cycle, not all strains were able to use citrate as the sole metabolite for growth (an indicator for a complete TCA cycle) (Dos Santos et al., 2017). Dos Santos et al. (2017) hypothesized that regulatory networks controlling the TCA cycle may explain the strain specific differences and discrepancies observed between studies over whether *Bordetella* has a functional TCA cycle. It is tempting to speculate that Ser/Thr/Tyr phosphorylation of the TCA cycle may be the regulator network responsible for these important metabolic differences as Ser/Thr/Tyr phosphorylation is known to be a key regulator of the TCA cycle in other bacteria and archaea (Macek et al., 2007; Macek et al., 2008; Esser et al., 2012), however further studies are required to confirm this.

### Ser/Thr Phosphorylation of BvgA May Affect DNA Binding Capability and Play a Key Role in the Adaptive Evolution of *Bordetella* From Environmental Species to Human Pathogenic Species

BvgAS is the primary regulator for over 550 genes in *Bordetella* (Moon et al., 2017). It is present in all classical and non-classical *Bordetella* species except *B. ansorpii* (Linz et al., 2016). Through phase variation, BvgA controls almost all *Bordetella* virulence genes, several metabolic and stress/protection response pathways and further regulates >25 different transcriptional regulators (Moon et al., 2017). It is well known that BvgA is phosphorylated at Asp54 by BvgS, its cognate sensor kinase. Asp54 is highly conserved in all bacterial two-component response regulators. Previously, it was suggested that Asp54 is the only site phosphorylated on BvgA (Boulanger et al., 2013) using phos-tag gels and that phosphorylation of Asp54 is both necessary and sufficient for virulence gene expression. Significantly however we discovered for the first time that BvgA is also highly phosphorylated on multiple Ser/Thr residues. The discovery of multiple new phosphorylated sites previously missed by Boulanger et al. (2013) and others may be due to our use of highly sensitive LC-MS/MS which can specifically identify phosphorylated amino acid residues as opposed to phos-tag gels which can only identify the presence of phosphorylation (as indicated by a shift in band size) but not which or how many amino acid residues are phosphorylated. Interestingly, in Boulanger et al. (2013) the deletion of BvgS, the mutation of Asp54 and the presence of Bvg modulating factors (50 mM MgSO_4_) were all found to eliminate BvgA phosphorylation in the phos-tag gel. This may suggest that additional Ser/Thr phosphorylation in BvgA may be dependent on the initial phosphorylation of Asp54.

In contrast to eukaryotes, multi-phosphorylated proteins in bacteria are rare and most phosphoproteins were mono-phosphorylated in *Bordetella*. BvgA however was highly phosphorylated with 7 phosphosites detected, 6 of which were located in the helix-turn-helix luxR domain for DNA binding. Multi-phosphorylated proteins in eukaryotes are common and act as “molecular switchboards” for signal fine tuning (Wang et al., 2001). The multi-phosphorylation of BvgA in the DNA-binding domain suggests that Ser/Thr phosphorylation may play an important role in stabilizing or destabilizing BvgA binding to DNA for further fine-tuning of virulence gene expression. Other two-component response regulators that were phosphorylated include BP0991 and BP1092 in *B. pertussis* and BPP0020 in *B. parapertussis*. All were phosphorylated at sites located in the helix-turn-helix domain for protein-DNA binding. This suggests that Ser/Thr phosphorylation may be a general mechanism for bacteria to control the ability of response regulators to bind DNA.

Evidence for Ser/Thr/Tyr phosphorylation in controlling the DNA-binding capacity of two-component response regulators such as BvgA has been observed in other Gram-positive pathogens including CovR in Group A and B *Streptococcus* (GAS/GBS) (Lin W. J. et al., 2009; Horstmann et al., 2014), GraR and VraR in *Staphylococcus aureus* (Fridman et al., 2013; Canova et al., 2014), WalR in *Bacillus subtilis* (Libby et al., 2015) and DosR in *Mycobacterium tuberculosis* (Chao et al., 2010). These interactions between two-component response regulators and Ser/Thr/Tyr phosphorylation were found to be important for virulence. Ser/Thr/Tyr phosphorylation can either antagonize or amplify the activity of response regulators. For example, CovR is the major response regulator that represses gene expression in GAS and GBS when Asp53 is phosphorylated by its cognate sensor kinase, CovS. However, phosphorylation of CovR at Thr65 was found to antagonize Asp53 and led to increased virulence gene expression by reducing/eliminating CovR’s ability to bind DNA (Lin W. J. et al., 2009; Horstmann et al., 2014). In contrast, phosphorylation of WalR at Thr101 in *B. subtilis* enhanced WalR activity and expression of WalR cell wall synthesis regulon (Libby et al., 2015). In Gram-negatives, the role of Ser/Thr/Tyr phosphorylation of response regulators has not yet been characterized, however there is limited evidence that phosphorylation of two-component system response regulators also occurs with Ser77 and Ser80 phosphorylated on ActR in *Sinorhizobium meliloti* (Liu et al., 2015). Our results provide further evidence that Ser/Thr phosphorylation of two-component response regulators is an important and unexplored regulatory mechanism in Gram-negative bacteria.

Multiple sequence alignment between all available *Bordetella* BvgA sequences revealed 4 phosphorylation sites in the helix-turn-helix luxR DNA binding domain that were exclusively present in all classical *Bordetella* species (including *Bordetella* genomospecies 6 whose closest ancestor is *B. bronchiseptica* (Spilker et al., 2014)) but absent in non-classical species. While BvgA is functionally interchangeable between the 3 classical species (Martínez De Tejada et al., 1996), functional differences between classical and non-classical BvgA has been demonstrated with *B. pertussis* and *B. holmesii* (Gerlach et al., 2004). When *B. holmseii* BvgA was introduced into *B. pertussis*, it was found that *B. holmesii* BvgA could be phosphorylated by *B. pertussis* BvgS. However, despite being phosphorylated at Asp54, *B. holmesii* BvgA could not bind to the virulence protomers in *B. pertussis* or lead to the expression of virulence genes (Horvat and Gross, 2009). Interestingly, when Horvat and Gross (2009) created a hybrid BvgA by replacing the *B. holmseii* BvgA DNA-binding domain with the *B. pertussis* BvgA DNA binding domain, DNA binding and virulence gene expression was restored. The mutational substitution of amino acids (not found to be phosphorylated in this study) in *B. homesii* BvgA did not restore promoter binding. This suggests that functional differences between classical and non-classical BvgA may be due to the ability of BvgA to undergo Ser/Thr phosphorylation at the helix-turn-helix luxR DNA-binding domain in classical *Bordetella* species. It also suggests that Ser/Thr phosphorylation of the DNA-binding domain in classical BvgA is essential for virulence gene expression. The speciation and adaptation of *Bordetella* from free-living environmental/opportunistic pathogens (non-classical bordetellae) to host-adapted pathogens (classical bordetellae) has been associated with progressive gene loss (Cummings et al., 2004; Linz et al., 2016), however it is also possible that pathogenic adaptation may also be associated with phosphoproteome changes. The Ser/Thr phosphorylation of BvgA may be a key event in the acquisition of virulence and evolution of the *Bordetella* genus into a human pathogen. This study identified several potential *Bordetella* Ser/Thr/Tyr kinases (BstK) and ongoing work is currently being performed to identify the kinase involved in Ser/Thr phosphorylation of BvgA and its role in virulence gene expression.

Interestingly, in the evolution of BvgAS in classical *Bordetella*, BvgS varied remarkably between species especially at the Venus flytrap 1 domain (VFT1) while in contrast, BvgA was highly conserved (Herrou et al., 2009). The highly conserved nature of BvgA provides further evidence of the potential importance of Ser/Thr phosphorylation in classical *Bordetella* species and is potentially a result of selection pressure to maintain phosphorylation.

### Ser/Thr/Tyr Phosphorylation of Key Virulence Factors and Its Role in Pathogenesis

Ser/Thr/Tyr phosphorylation was found on T3SS (BtrV and BcrH2) and iron acquisition (BfrH, AlcA and AlcC) proteins. The T3SS is an important virulence determinant used by bacterial pathogens to deliver effectors to host cells. The T3SS is present in all classical *Bordetella* species and absent in most non-classical *Bordetella* species (except *B. ansorpii*) (Linz et al., 2016). BtrV is an important anti-anti sigma factor involved in the regulation of T3SS and forms a partner switching complex with two other proteins, BtrU and BtrW. When BtrV is unphosphorylated (or dephosphorylated by BtrU), it binds to BtrW and inhibits type III secretion. Upon phosphorylation by BtrW, BtrV dissociates and type III secretion can occur (Kozak et al., 2005). BtrV was previously found by Kozak et al. (2005) to be phosphorylated at the conserved Ser55 site, however the authors could not rule out phosphorylation of the adjacent site, Ser54. In this study, we detected the monophosphorylation of BtrV at two sites, Ser55 and Ser54, with Ser55 being the predominant site. This study also detected the phosphorylation of BcrH2 at Ser148. BcrH2 is a chaperonin protein that transports the pore forming proteins BopB-BopD from the bacterial cytoplasm (Nogawa et al., 2004). The role of BcrH2 phosphorylation is currently unknown, however it may affect protein turnover or the stabilization of BcrH2 to BopB-BopD.

Iron acquisition is an essential trait for all pathogenic bacteria. This study found three proteins associated with iron acquisition to be phosphorylated including two (AlcA and AlcC) belonging to the alcaligin synthesis pathway. Alcaligin is the primary siderophore produced by *Bordetella* species. The pathway is present in all classical *Bordetella* species but absent in most non-classical *Bordetella* species (except *B. holmesii*) (Linz et al., 2016). Phosphorylation of key enzymes in this pathway may be an important regulator for iron uptake and virulence in the classical bordetellae group.

Finally, tyrosine phosphorylation has been previously linked to bacterial pathogenicity (Ge and Shan, 2011) with pathogens such as enterohemorrhagic *E. coli* (Hansen et al., 2013), *Shigella flexneri* (Standish et al., 2016), *Helicobacter pylori* (18.5% pTyr) (Ge et al., 2011), *Pseudomonas aeruginosa* (14.5% pTyr) (Ravichandran et al., 2009) and *Klebsiella pneumoniae* (25.8% pTyr) (Lin M.-H. et al., 2009) (**Supplementary Table 7**) having high abundances of pTyr sites. It was found that *P. aeruginosa* (14.5% had higher pTyr sites compared to its non-pathogenic counterpart *P. putida* (7.5%) (Ravichandran et al., 2009). However, this study did not identify widespread tyrosine phosphorylation or a difference in the proportion of pTyr sites between the 3 classical species despite *B. pertussis* considered to have a higher pathogenic potential compared to its ancestor *B. bronchiseptica* and causes greater disease severity compared to *B. parapertussis*. It is possible that differences in pTyr may exist between classical pathogenic and non-classical environmental/opportunistic *Bordetella* species. Additional enrichment of pTyr using specific antibodies may also be needed to completely elucidate the role of tyrosine phosphorylation in *Bordetella* virulence. This suggests that some of the potential Hanks-type Ser/Thr kinases identified in this study may be dual specificity protein kinases (DSPK) that can phosphorylate Ser/Thr and Tyr. Examples of DSPK have been identified in *Bacillus anthracis* and *M. tuberculosis* (Arora et al., 2012; Kusebauch et al., 2014).

## Conclusion

**Figure 9:**
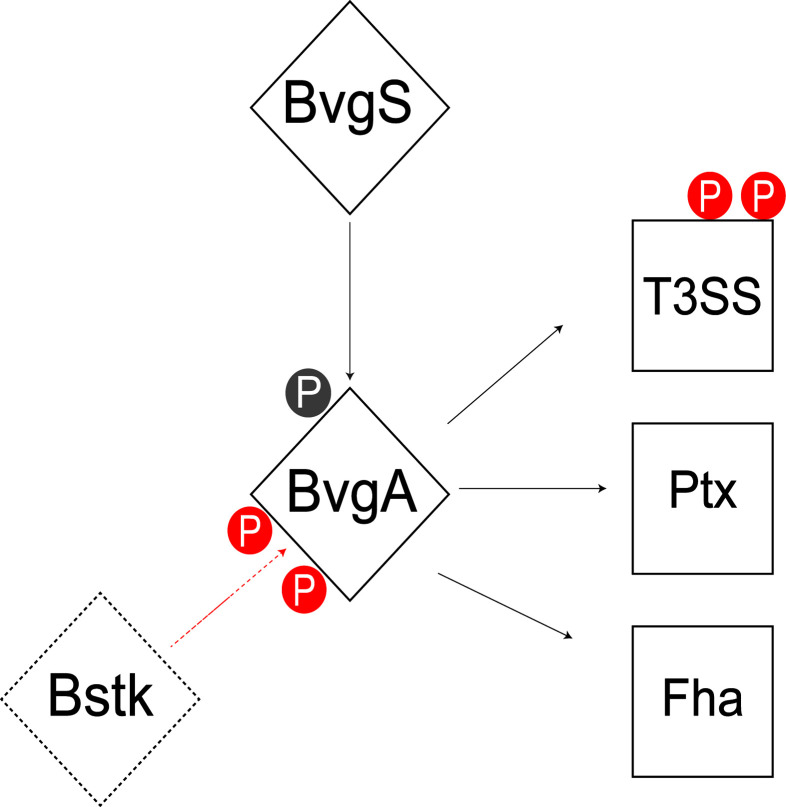
Summary of the Bvg phosphorylation model in *Bordetella*. BvgA is phosphorylated by BvgS at Asp54 (grey) and one or more unknown Bstk (red) during the Bvg+ phase. BvgA regulates the expression of several virulence factors including the type III secretion system (T3SS), pertussis toxin (Ptx) and filamentous hemagglutinin (Fha). Some of these BvgA-regulated virulence factors such as T3SS are also phosphorylated at Ser/Thr/Tyr sites (red).

This study analyzed the Ser/Thr/Tyr phosphoproteome of 3 classical *Bordetella* species and identified 70 unique phosphorylated proteins, of which, 41% were phosphorylated in two or more species. Core metabolic pathways in *Bordetella* were significantly enriched for phosphorylated proteins including gluconeogenesis, the TCA cycle, amino acid and nucleotide synthesis. This study found that serine/threonine phosphorylation of virulence pathways may be an important virulence determining factor separating classical from non-classical *Bordetella* species. Interestingly, BvgA was found to be heavily phosphorylated by a yet unknown Bstk, especially at the helix-turn-helix luxR domain with 7 different phosphosites identified. This indicates a potential role for Ser/Thr/Tyr phosphorylation to stabilize/destabilize BvgA binding to DNA for virulence regulation (**Figure 9**). Additional phosphorylation sites were also identified in BvgA-regulated proteins such as the T3SS, possibly providing another layer of control.

As the aim of this study was to provide the first snapshot of the phosphoproteome in classical *Bordetella* species, it is likely that under different growth stages and conditions such as other Bvg phases, iron limitation and biofilm formation etc., other important phosphorylation events for biology and virulence will be identified. Further characterization of the phosphoproteome in non-classical bordetellae especially environmental associated *Bordetella* species will also uncover the role of phosphorylation in the evolution of the *Bordetella* genus toward a host-adapted pathogen. In conclusion, this study provides the first insight into the Ser/Thr/Tyr phosphoproteome of classical *Bordetella* species and its role in *Bordetella* biology and virulence.

## Data Availability Statement

The datasets presented in this study can be found in online repositories. The names of the repository/repositories and accession number(s) can be found below: http://www.proteomexchange.org/, PXD020866.

## Author Contributions

LL: conceptualization, methodology, investigation, formal analysis, writing—original draft preparation, and visualization. LZ: methodology, formal analysis, validation, and writing—review and editing. SK: software, formal analysis, and writing—review and editing. MR: methodology, formal analysis, validation, and writing—review and editing. RL: conceptualization, methodology, supervision, formal analysis, resources, funding acquisition, and writing—review and editing. All authors contributed to the article and approved the submitted version.

## Funding

The funding for this project was provided by the National Health and Medical Research Council of Australia (NHMRC).

## Conflict of Interest

The authors declare that the research was conducted in the absence of any commercial or financial relationships that could be construed as a potential conflict of interest.

## Supplementary Material

Supplementary Table 1 List of Ser/Thr/Tyr phosphorylated proteins, peptides and sites identified in *B. pertussis* L1423 with a localization probability (A-score) ≥95%.

Supplementary Table 2 List of Ser/Thr/Tyr phosphorylated proteins, peptides and sites identified in *B. parapertussis* 12822 with a localization probability (A-score) ≥95%.

Supplementary Table 3 List of Ser/Thr/Tyr phosphorylated proteins, peptides and sites identified in *B. bronchiseptica* RB50 with a localization probability (A-score) ≥95%.

Supplementary Table 4 Common phosphorylated proteins identified in two or more *Bordetella* species. Localization probability below the cut-off are indicated.

Supplementary Table 5 List of unique phosphoproteins detected in *Bordetella* proteomic studies.

Supplementary Table 6 List of significantly functionally enriched clusters of phosphorylated proteins identified with STRING v11.

Supplementary Table 7 Comparison of the amount of starting proteins digested and the number of Ser/Thr/Tyr phosphoproteins detected in this study with previous bacterial phosphoproteomic studies.
